# Identification of the protein coding capability of coronavirus defective viral genomes by mass spectrometry

**DOI:** 10.1186/s12985-023-02252-3

**Published:** 2023-12-07

**Authors:** Ching-Hung Lin, Feng-Cheng Hsieh, Chien-Chen Lai, Wei-Chen Wang, Cheng-Yu Kuo, Chun-Chun Yang, Hsuan-Wei Hsu, Hon-Man-Herman Tam, Cheng-Yao Yang, Hung-Yi Wu

**Affiliations:** 1 Graduate Institute of Veterinary Pathobiology, College of Veterinary Medicine, National Chung Hsing University, Taichung, 40227 Taiwan; 2 Institute of Molecular Biology, College of Life Sciences, National Chung Hsing University, Taichung, 40227 Taiwan; 3 Department of Veterinary Medicine, College of Veterinary Medicine, National Chung Hsing University, Taichung, 40227 Taiwan

**Keywords:** Coronavirus, Defective viral genome, Protein coding, Gene expression, Pathogenesis

## Abstract

During coronavirus infection, in addition to the well-known coronavirus genomes and subgenomic mRNAs, an abundance of defective viral genomes (DVGs) can also be synthesized. In this study, we aimed to examine whether DVGs can encode proteins in infected cells. Nanopore direct RNA sequencing and liquid chromatography-tandem mass spectrometry (LC–MS/MS) analysis were employed. With the protein databases generated by nanopore direct RNA sequencing and the cell lysates derived from the RNA–protein pull-down assay, six DVG-encoded proteins were identified by LC–MS/MS based on the featured fusion peptides caused by recombination during DVG synthesis. The results suggest that the coronavirus DVGs have the capability to encode proteins. Consequently, future studies determining the biological function of DVG-encoded proteins may contribute to the understanding of their roles in coronavirus pathogenesis and the development of antiviral strategies.

## Introduction

Coronavirus (CoV), which has the largest known viral RNA genome of ~ 30 kilobases (kb), is a single-stranded, positive-sense RNA virus [1, 2]. CoVs are critical pathogens of humans and animals that can lead to widespread and costly diseases, such as COVID-19, which is caused by severe acute respiratory syndrome coronavirus 2 (SARS-CoV-2) [3, 4]. The genome can encode a polyprotein that is then cleaved into 15–16 nonstructural proteins (nsps). During coronavirus infection, in addition to the coronavirus genomes, a nested set of subgenomic mRNAs (sgmRNAs) are also produced from which structural and accessory proteins are translated [5]. The recent studies by nanopore direct RNA sequencing suggest that during coronavirus infection, in addition to the well-known coronavirus genomes and sgmRNAs, defective viral genomes (DVGs) are synthesized in abundance [6–10]. Although the mechanisms by which the DVGs are synthesized remain unclear, it is believed that they may be synthesized by a copy-choice template-switching recombination process during coronavirus replication [11–13].

Accumulated data have shown that DVGs are associated with pathogenesis of RNA viruses [14, 15]. For example, highly pathogenic influenza virus isolates with an impaired ability to produce DVGs lose their function to induce innate immunity and thus cause more severe flu symptoms [16]. In addition, the DVGs in RNA viruses such as paramyxovirus and Ebola virus also have been shown to be associated with the establishment of virus persistence [17, 18]. Consequently, DVGs may play important roles in the pathogenicity and the subsequent outcome of diseases. On the other hand, because some of the DVGs contain open reading frames, it is therefore speculated that they may express novel proteins and have functions affecting viral replication and pathogenesis. Based on the definition that DVGs are viral genomes which contain small to large internal substitutions, deletions, and/or insertions, and thus unable to produce the original viral proteins, but maintain their replication potential [19], it has been suggested that the long-term transmission of dengue virus is associated with the protein encoded by the dengue virus DVG [20]. In addition, it has been shown that a novel protein encoded by a DVG derived from the polymerase basic 2 protein (PB2) segment of influenza virus can induce type I IFN and is associated with disease severity in a mouse model [21]. Further study demonstrates that the DVG-encoded protein in influenza virus can restrict viral replication through modulation of the antiviral host gene response and thus is a virulence factor [22]. Therefore, due to the diverse structures of DVGs, in addition to DVGs, the DVG-encoded proteins may also have various impacts on the viral replication and thus pathogenesis. In coronavirus, DVGs have been suggested to interfere with the replication of some coronaviruses [11] and to be involved in the modulation of host IFN responses [23], contributing to the pathogenesis. In addition, DVGs have been demonstrated to be antivirals that inhibit SARS-CoV-2 replication [24, 25]. However, the knowledge on protein coding capability, the function of DVG-encoded proteins and their roles in coronavirus replication and pathogenesis remain limited.

The characteristics of coronavirus DVGs have been previously identified [9, 26]. The studies suggest that the synthesis of DVGs is reproducible under regular infection environment, but the amounts and the species of DVGs are altered under different infection conditions [9, 26]. In the current study, we extended the observations by determination of their protein coding capability. For this, the protein databases generated from the results of nanopore direct RNA sequencing of bovine coronavirus (BCoV) were used as references for liquid chromatography-tandem mass spectrometry (LC-MS/MS) analysis to validate the protein coding capability of BCoV DVGs. The limitations and the biological significance of the study are discussed.

## Materials and methods

### Viruses and cells

The plaque-purified Mebus strain of bovine coronavirus (BCoV) (GenBank: U00735.2) was obtained from David A. Brian (University of Tennessee, TN) and grown in human rectal tumor (HRT)-18 cells. HRT-18 cells were obtained from David A. Brian (University of Tennessee, TN). The aforementioned cells were grown in Dulbecco’s modified Eagle’s medium (DMEM) supplemented with 10% fetal bovine serum (HyClone, UT, USA) at 37 °C with 5% CO_2_ as previously described.

### Establishment of DVG data bases by nanaopore direct RNA sequencing

The detailed methods for nanaopore direct RNA sequencing for BCoV were described previously [9, 26]. To establish the DVG databases, total cellular RNA collected from BCoV-infected HRT-18 cells at 24 h postinfection was extracted by TRIzol (Thermo Fisher Scientific, Waltham, USA) and 500 ng of poly(A)-containing RNA was used for library preparation according to the manufacturer’s instructions (SQK-RNA001, Oxford Nanopore Technologies). Two biological replicates were performed for nanopore direct RNA sequencing. The prepared library was loaded onto an ONT FLO-MIN106D flow cell, and sequencing was conducted for 24 h on a MinION device (Oxford Nanopore Technologies). The data collected from the MinION device were base called by Guppy (v5.0.11) with a q-score of 7. The base-called data were first mapped to the host genome (HRT-18 cells: GRCh38) using Minimap2 (v2.17-r941) 1 with the parameters “-k 14 -w 1 --splice -u n --MD -a -t 6 --secondary = no” and then mapped to virus virus genome (BCoV: U00735.2) using Minimap2 (v2.17-r941) with the parameters “-Y -k 8 -w 1 --splice -g 30000 -G 30000 -F 40000 -N 32 --splice-flank = no --max-chain-skip = 40 -u n --MD -a -t 24 --secondary = no” to generate SAM files. The host genome was not removed before mapping to virus genome. The SAM files generated with the parameters described above were polished by TranscriptClean (v2.0.3) and then transformed into BAM files by SAMtools (v1.15). The resulting BAM files were further processed by bedtool (2.28) to generate BED files. The BED files containing nucleotide sequences of coronavirus transcripts were converted to amino acids via Biostrings (2.68) in R (v4.1.2). The criteria for the conversion were as follows. First, the first two complete open reading frames (ORFs) from the DVG were selected. Second, the ORF contained the start codon (ATG) and the stop codons (TAA/TAG/TGA). Third, the translated protein contained more than 10 amino acids. Consequently, Protein reference databases derived from the nanopore direct RNA sequencing of BCoV were then established (https://osf.io/cm7z6/; file path:Data_analysis/(4)Mass_spectrometer_analysis/BCoV_cell_ORF_DVG_nanopore.xls).

### Liquid chromatography-tandem mass spectrometry (LC–MS/MS) analysis

For identification of DVG-encoded proteins, HRT-18 cells were infected with BCoV at an MOI of 0.1, and cell lysates were collected at 24 h postinfection. The collected cell lysates were sent directly for LC–MS/MS analysis or for RNA–protein pull-down assay followed by LC–MS/MS analysis. For the RNA–protein pull-down assay [27], a DNA template containing a bulge stem loop and pseudoknot from the BCoV 3’ UTR was used for in vitro transcription with T7 polymerase (Promega) in the presence of a biotin-UTP labeling NTP mixture (Roche), as recommended by the manufacturer. After purification, 10 μg of biotinylated RNA was incubated with 2 mg of cell lysates in TE buffer. After incubation at room temperature for 30 min, a streptavidin suspension (MagQu) was added to the mixture and incubated for 30 min at room temperature followed by five washes with lysis buffer. The protein-associated beads were eluted with SDS–PAGE loading buffer and subjected to mass spectrometry analysis by Dr. Chien-Chen Lai at the Institute of Molecular Biology, National Chung Hsing University, Taichung, Taiwan.

For the treatment of cell lysates for LC–MS analysis [28–30], the cell lysates used for SDS–PAGE were mixed with dye and heated for 5 min at 95 °C. The denatured proteins were separated on 11% resolving gel with 5% stacking gel, and separated on 28% resolving gel with 11% stacking gel, separately. After electrophoresis, gels were maintained in a fixation buffer containing 30% ethanol and 10% acetic acid. Next, silver staining was applied, and the stained gels were scanned using a Perfection V750 Pro scanner (Epson, USA). The in-gel digestion method was described by Chien et al. [29]. The bands were collected and then cut into small pieces. The gel pieces were washed with 50 mM ammonium bicarbonate (ABC) buffer and 50% acetonitrile (ACN)/100 mM ABC buffer and dehydrated with 100% ACN. After vacuum drying, reduction buffer was added, and samples were incubated for 1 h at 56 °C. This step was followed by the addition of alkylation buffer and incubation for 30 min at 37 °C in the dark. The gel pieces were then washed with 100 mM ABC/50% ACN, dehydrated with 100% ACN, and vacuum dried. The gels were then incubated with 10 ng/μL trypsin in 50 mM ABC for 16 h at 37 °C. After trypsin digestion, an equivalent volume of peptide extraction solution (50% ACN/0.1% FA) was added. Next, the supernatant was transferred into a new collection tube, and peptide extraction solution was added again to gel pieces under heating for 1 h at 37 °C. Finally, all the tryptic peptide extracts were merged, vacuum dried and stored at − 20 °C.

For nano-LC/MS/MS analysis, each sample was dissolved in 0.1% FA solution and then separated through UPLC (Thermo-Dionex, Sunnyvale, CA, USA). Samples were trapped and concentrated in a nanoViper C18 trap column (100 μm × 20 mm, 100 Å, 5 μm, Thermo Fisher) with a flow rate of 10 μL/min that was connected to a nanoViper C18 analytical column (75 μm × 250 mm, 100 Å, 2 μm, Thermo Fisher) with a flow rate of 0.3 μL/min for separation at an oven temperature of 35 °C. A binary gradient system was used, consisting of mobile phases A and B, which were 0.1% FA and 0.1% FA in ACN, respectively. The gradient was programmed as follows: 0–4.5 min, 5% B; 4.5–31 min, 5–35% B; 31–32 min, 35–90% A; 32–52 min, 90% B; 52–53 min, 90–5% B; 53–70 min, 5% B. The injection volume was 5 μL. The TripleToF 6600 (SCIEX, Framingham, MA) was operated in the positive ion mode with an ion spray floating voltage of 2800 V. The interface heater temperature was set at 150 °C. Both the nebulizer gas and curtain gas were nitrogen, which was used at 25 and 20 psi, respectively. The declustering potential was set at 80 V. The accumulation time was 250 msec. Data-dependent acquisition was scanned in the range of m/z 350 to 1,250 for the collection of MS/MS spectra for the 30 most abundant precursor ions, with two to four charge states (counts > 100 cps). The exclusion of former target ions was set for 12 s after 1 occurrence, and the mass tolerance was set to 50 mDa. The MS/MS spectra were accumulated for 80 msec over the range m/z 65 to 1,800 with rolling collision energy. To ensure mass accuracy and sensitivity, 25 fmol/μL β-galactosidase (SCIEX) was used for quality control.

All the spectra generated by MS were searched thoroughly against the *Homo sapiens* database (https://www.uniprot.org/uniprotkb?query=homo+sapiens) downloaded from UniProt (https://www.uniprot.org/) and the in-house database derived from the nanopore direct RNA sequencing of BCoV using the Mascot Server (version 2.3.0, Matrix Science). The search parameters were as follows: type of search: MS/MS ion search; fixed modifications: carbamidomethyl (C); variable modifications: deamidated (NQ), oxidation (HW), oxidation (M); mass values: monoisotopic; protein mass: unrestricted; peptide mass tolerance: ± 0.03 Da; fragment mass tolerance: ± 0.05 Da; max missed cleavages: 1; and instrument type: ESI-QUAD-TOF. The databases of identified proteins by LC-MS/MS analysis are as follows. The databases for DVG-encoded proteins (total cell lysates) using protein reference databases derived from nanopore direct RNA sequencing are deposited at https://osf.io/cm7z6/; file path: Data_analysis/(4) Mass_spectrometer_analysis/BCoV-infected_HRT_total_cell_lysate/BCoV-infected_HRT_total_cell_lysate. The databases for DVG-encoded proteins (RNA-protein pull-down lysates) using protein reference databases derived from nanopore direct RNA sequencing are deposited at https://osf.io/cm7z6/; file path: Data_analysis/(4) Mass_spectrometer_analysis/BCoV-infected_HRT_with_RNA_pull-down_cell_lysate/(A) BCoV_DVG_database_result/BCoV with RPDCL by DVG. The databases for encoded proteins from human cells (RNA-protein pull-down lysates) using human protein sequences as reference databases are deposited at https://osf.io/cm7z6/; file path: Data_analysis/(4) Mass_spectrometer_analysis/BCoV-infected_HRT_with_RNA_pull-down_cell_lysate/(B) human_database_result /BCoV with RPDCL by human.

## Results

### Fusion peptides as markers to determine the proteins encoded by coronavirus DVGs

According to our database established by nanopore direct RNA sequencing (https://osf.io/cm7z6/), bovine coronavirus (BCoV) DVGs contain open reading frames (ORFs) of various lengths from one or more portions of ORFs in the full-length genome due to recombination during virus replication. These DVGs with diverse genome structures may lead to the synthesis of in-frame, out-of-frame, or fusion proteins when compared with the original ORFs in the full-length genome. Accordingly, to identify the proteins encoded by DVGs bearing diverse ORFs, liquid chromatography-tandem mass spectrometry (LC–MS/MS) analysis was employed. As described above, the diverse genome structures of DVGs may encode in-frame proteins that have the same amino sequences as canonical genome- and sgmRNA-encoded proteins. Consequently, if the amino acid sequences of the peptides determined by LC–MS/MS analysis contain exactly the same in-frame amino acid sequences as those of canonical genome- and sgmRNA-encoded proteins, these peptides cannot be used as markers to determine whether the identified proteins are encoded from coronavirus DVGs. In contrast, if the peptides contain discontinuous in-frame amino acid sequences derived from different portions of amino acid sequences from canonical genome- or sgmRNA-encoded proteins or contain out-of-frame amino acid sequences, they are considered fusion peptides encoded by DVGs caused by recombination of the viral genome. Therefore, these fusion peptides can be used as markers to identify the proteins encoded by coronavirus DVGs.

### DVG-encoded proteins with featured fusion peptides were identified

**Fig. 1:**
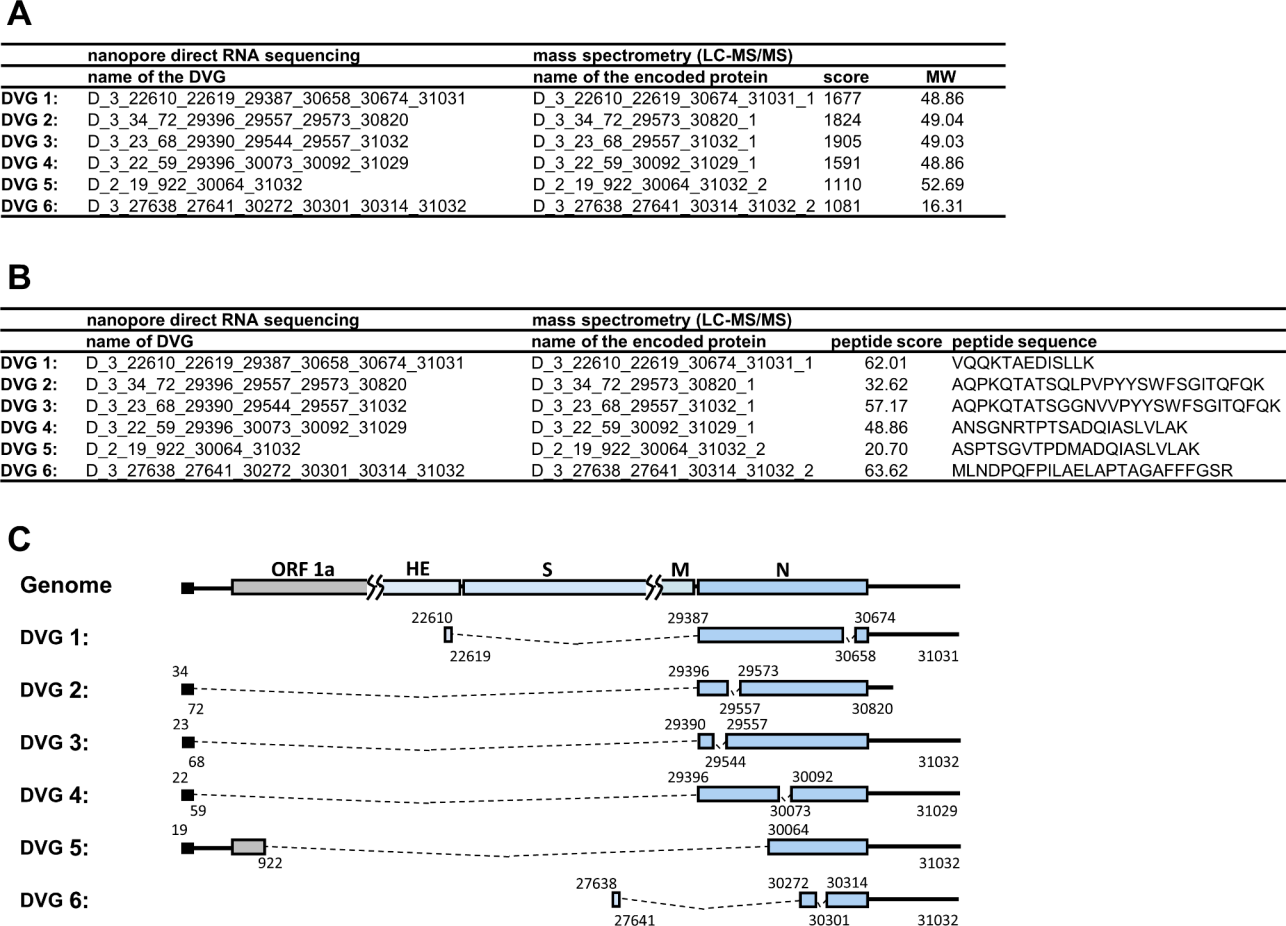
Identification of DVG-encoded proteins by LC–MS/MS analysis. (**A**) The protein scores and molecular weights (MWs) derived from LC–MS/MS analysis for DVG-encoded proteins. (**B**) The amino acid sequences and scores of featured fusion peptides that specifically match the amino acid sequences of proteins encoded from the DVGs. (**C**) The structures of the DVGs (determined by nanopore direct RNA sequencing) from which proteins are encoded (determined by LC–MS/MS analysis) shown in (A) and (B). The numbers shown in each DVG structure are the nucleotide positions at which recombination occurs. The dashed line indicates the truncated genome in DVG

A total of 145,015 DVG species were identified in the two biological replicates for nanopore direct RNA sequencing, and 189,221 amino acid sequences were converted to protein reference databases (https://osf.io/cm7z6/; file path: Data_analysis/(4) Mass_spectrometer_analysis/BCoV_cell_ORF_DVG_nanopore.xlsx) according to the three criteria described in Materials and methods. The databases were then used as references for liquid chromatography-tandem mass spectrometry (LC–MS/MS) analysis to validate the synthesis of BCoV DVG-encoded proteins. A total of 34,104 protein species were identified by LC–MS/MS analysis (https://osf.io/cm7z6/; file path: Data_analysis/(4) Mass_spectrometer_analysis/BCoV-infected_HRT_total_cell_lysate/BCoV-infected_HRT_total_cell_lysate ). However, none of the featured fusion peptides that can be used to represent actual DVG-encoded proteins were detected. These results were not surprising because there were so many species of DVGs in the cells, and thus, the amount of each DVG-encoded protein (especially proteins with the featured fusion peptides) in a fixed amount of cell lysate may not be sufficient to be detected by LC–MS/MS analysis. Consequently, it was speculated that with enrichment processes such as RNA–protein pull-down assays, by which the proteins binding to RNA can be isolated, the amount of each protein species can be increased and thus detected by LC–MS/MS, although fewer DVG-encoded protein species may be detected. As a result, 34,056 protein species were identified by LC–MS/MS analysis (https://osf.io/cm7z6/; file path: Data_analysis/(4) Mass_spectrometer_analysis/BCoV-infected_HRT_with_RNA_pull-down_cell_lysate/(A) BCoV_DVG_database_result/BCoV with RPDCL by DVG), but only 7 DVG-encoded proteins with featured fusion peptides were identified. Among the 7 identified peptides, LFLYGGR was identified with three consecutive y ions in both spectra. However, because (i) based on the LC–MS/MS analysis, proteins with a score higher than 41 (*p* < 0.05) can be considered a confident identification and (ii) in addition to peptide LFLYGGR, there were no other peptides detected, leading to the score [17] of the DVG-encoded protein being lower than 41, we could only confirm the existence of peptide LFLYGGR, but not that of DVG-encoded protein. Consequently, only 6 featured fusion peptides (Fig. 1) that are derived from DVGs and can actually represent DVG-encoded proteins were detected with the enrichment process (https://osf.io/cm7z6/; file path: Data_analysis/(4) Mass_spectrometer_analysis/BCoV-infected_HRT_with_RNA_pull-down_cell_lysate/(A) BCoV_DVG_database_result/BCoV with RPDCL by DVG). In addition, the 6 featured fusion peptides were not identified using human protein sequences as reference (https://osf.io/cm7z6/; file path: Data_analysis/(4) Mass_spectrometer_analysis/BCoV-infected_HRT_with_RNA_pull-down_cell_lysate/(B) human_database_result /BCoV with RPDCL by human).

**Fig. 2:**
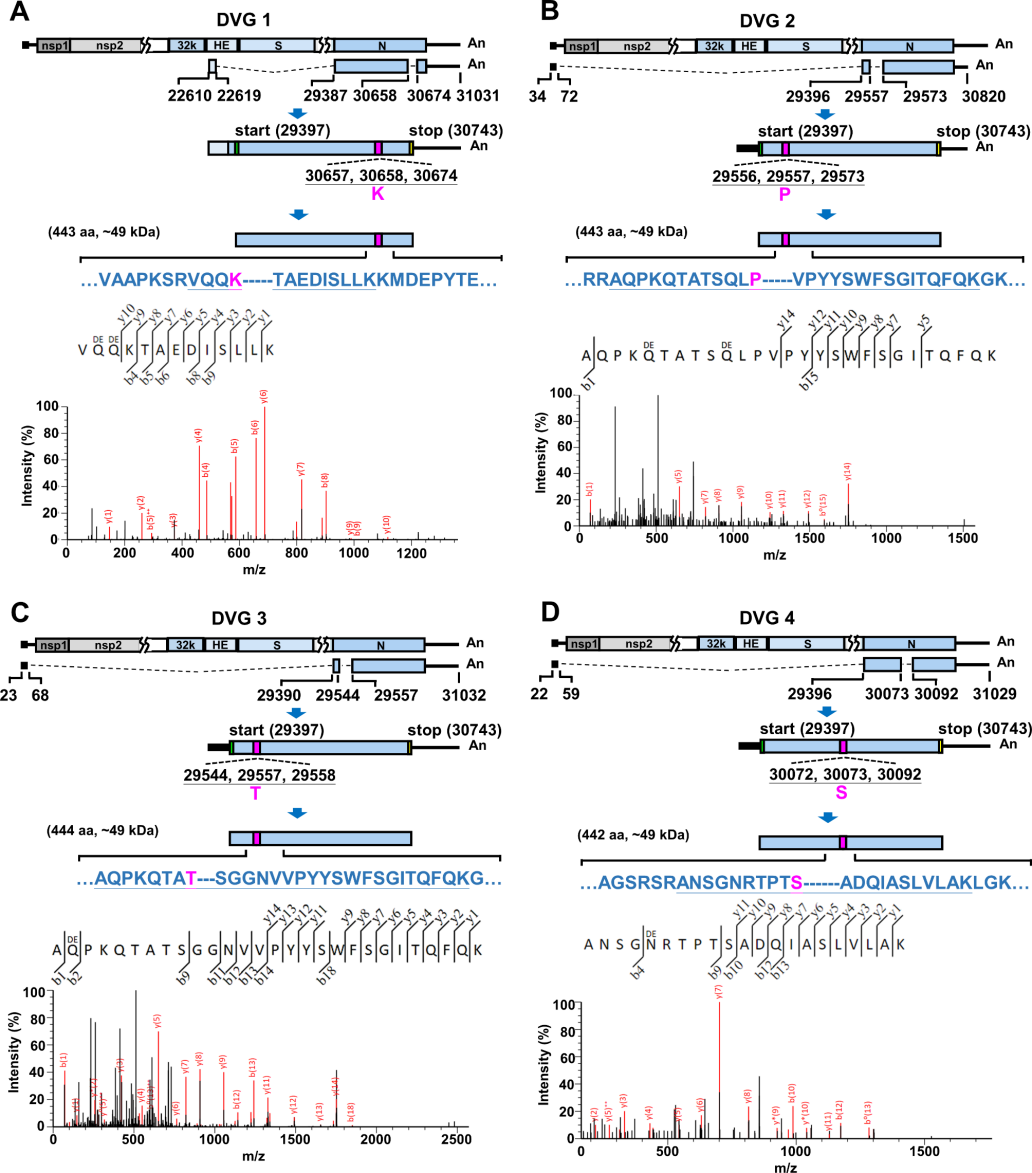
Identification of fusion peptide fragments derived from proteins encoded by DVGs 1–4 based on LC–MS/MS analysis. (**A**)-(**D**) The genome structures and open reading frames (ORFs) of the DVGs 1–4 (upper panels). The numbers shown in each DVG structure are the nucleotide positions at which the recombination occurs. The amino acid sequences containing mutations (indicated in pink) or/and deletions (indicated by dashes) which match to these identified by LC-MS/MS analysis (lower panel) are underlined. The values of m/z for DVGs 1–4 are as follows: 492.2727 (DVG 1); 1035.1818 (DVG 2); 1031.1800 (DVG 3); 705.7044 (DVG 4). aa, amino acid

**Fig. 3:**
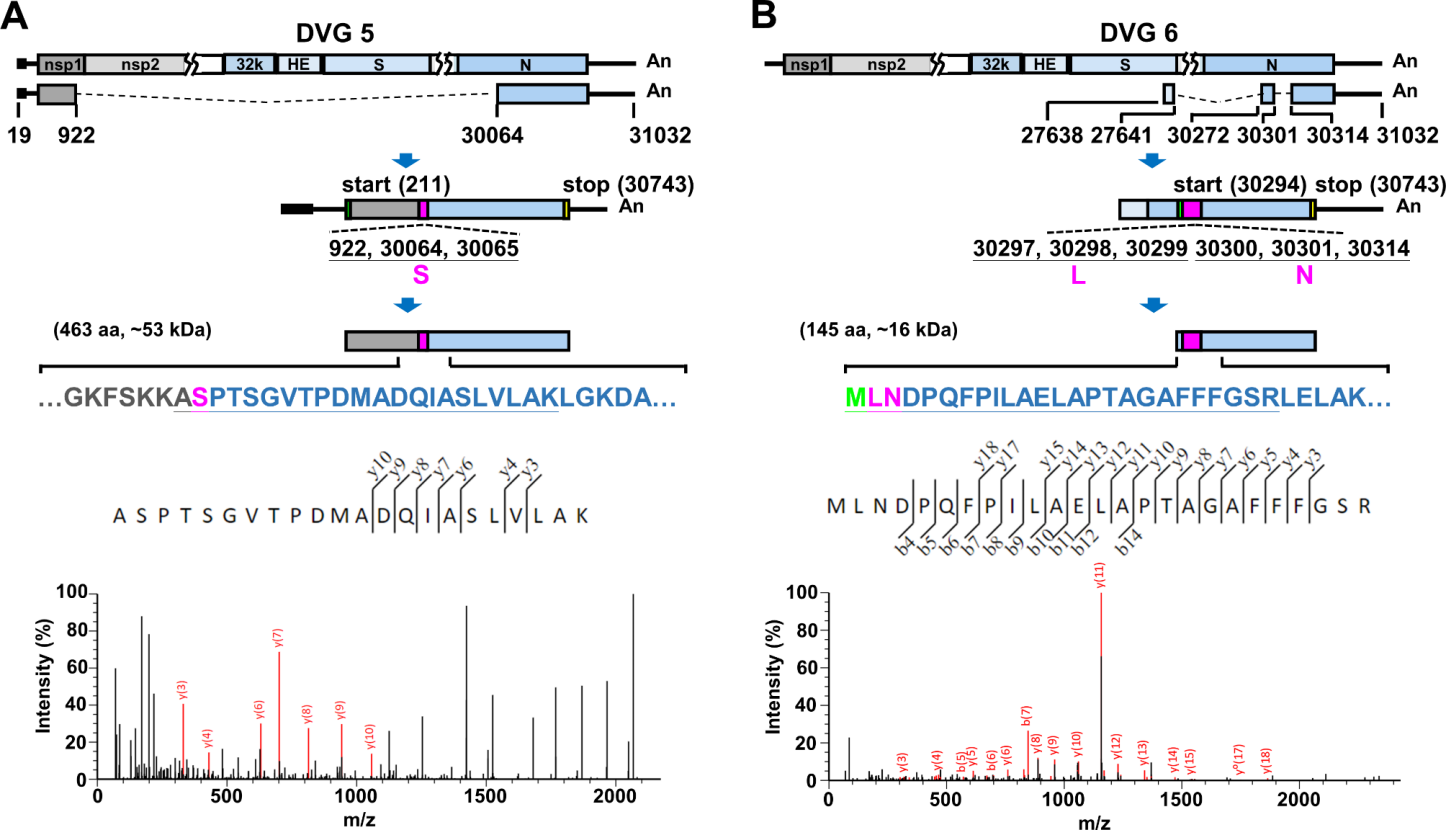
Identification of fusion peptide fragments derived from proteins encoded by DVGs 5–6 based on LC-MS/MS analysis. (**A**)-(**B**) The genome structures and open reading frames (ORFs) of the DVGs 5–6 (upper panels). The numbers shown in each DVG structure are the nucleotide positions at which the recombination occurs. The amino acid sequences containing mutations (indicated in pink) or/and deletions (indicated by dashes) which match to these identified by LC-MS/MS analysis (lower panel) are underlined. The amino acid M (indicated in green) in DVG 5 is derived from 30,294, 30,295 and 30,296. The values of m/z for DVGs 5–6 are as follows: 724.7131 (DVG 5); 904.1380 (DVG 6). aa, amino acid

As shown in Fig. 2A, DVG 1, which consisted of part of the HE protein-encoding gene (nucleotides 22610–22619), a gene upstream of the nucleocapsid (N) protein-encoding gene (nucleotides 29387–29396), part of the N protein-encoding gene (nucleotides 29397–30658) and part of the N protein-encoding gene with a 3’ UTR (nucleotides 30,674–31,031), was predicted to encode a protein with a fusion peptide. The predicted protein (443 amino acids) encoded from the DVG contained three parts of the N protein resulting from recombination and deletion within the N protein-encoding gene, including part of the in-frame N protein (amino acids 1-420); the recombination-derived amino acid K 421 resulting from nucleotides 30,657, 30,658 and 30,674; and part of the in-frame N protein (amino acids 422–443). In addition to the recombination-derived amino acid K, the deletion between nucleotides 30,659–30,673 also led to the deletion of 5 amino acids. Mass spectrometry (Fig. 2A, lower panel) identified the featured fusion peptide fragment with amino acid sequence VQQKTAEDISLLK, which matched amino acids 418–430 resulting from mutation and deletion (Fig. 2A, upper panel) within the DVG-encoded protein. Such fusion peptide fragments with the recombination feature were also identified in other DVG-encoded proteins by LC–MS/MS analysis, and are illustrated in Figs. 2 and 3. It is therefore concluded that coronavirus DVGs have the capability to encode proteins.

## Discussion

In the current study, nanopore direct RNA sequencing and liquid chromatography-tandem mass spectrometry (LC–MS/MS) analysis were employed to examine whether DVGs can encode proteins in infected cells. With the protein databases generated by nanopore direct RNA sequencing, six DVG-encoded proteins were identified by LC–MS/MS based on the featured fusion peptides caused by recombination during DVG synthesis. The limitations and the biological significance of the study are discussed.

Below, we explain why 34,104 (by total cell lysates) and 34,056 (by cell lysates derived from RNA–protein pull-down assay) protein species were identified by LC–MS/MS analysis. First, coronavirus DVGs are recombination products and thus contain ORFs of various lengths from one or more portions of ORFs in the full-length genome. As a result, many DVG species (145,015) are identified by nanopore direct RNA sequencing, and thus, many potential DVG-encoded protein sequences (189,221) can be used as protein reference databases for LC–MS/MS. Second, the diverse genome structures of DVGs may encode in-frame peptides that have the same amino sequences as those encoded from the full-length genome. Consequently, if the peptides determined by LC–MS/MS analysis match the amino acid sequences of the DVG-encoded proteins and the protein scores are higher than 41, the DVG-encoded protein species can be identified based on the provided protein reference databases. Consequently, many DVG-encoded protein species (34,104 from total cell lysates, and 34,056 from cell lysates by RNA–protein pull-down assay) were identified by LC–MS/MS analysis. However, this may lead to false-positive results because the peptides that match the amino acid sequence of DVG-encoded proteins may also be encoded from the full-length coronavirus genome, as described above, and thus cannot be used as markers to determine whether the identified proteins are encoded by coronavirus DVGs. That is also the reason why we propose that if the peptides contain discontinuous in-frame amino acid sequences derived from different portions of amino acid sequences from full-length genome-encoded proteins or contain out-of-frame amino sequences, the peptides are fusion peptides encoded from DVGs because DVGs are synthesized by recombination of the viral genome. Therefore, these fusion peptides can be used as markers to identify the proteins actually encoded by coronavirus DVGs. Consequently, 6 DVG-encoded proteins were identified through the identification of 6 fusion peptides, as shown in Figs. 1, 2 and 3.

In addition, because the read number for the 6 DVGs is low (only 1), whether there is a correlation between the abundance of DVGs identified by nanopore direct RNA sequencing and that of their encoded proteins identified by LC-MS/MS remains unknown. Our explanation for the results is as follows. Because coronavirus DVGs are recombination products and thus contain ORFs of various lengths from one or more portions of ORFs derived from the full-length genome, the diverse genome structures of DVGs may encode in-frame peptides that have the same amino sequences as those encoded from the full-length genome. Consequently, if the peptides determined by LC-MS/MS analysis match the amino acid sequences of DVG-encode proteins and the protein scores are higher than 41, the DVG-encoded protein species are identified based on the provided protein reference databases. However, the peptides which match the amino acid sequence of DVG-encoded proteins may also be encoded from full-length coronavirus genome, and thus we cannot determine whether the identified peptides and thus the proteins are encoded from coronavirus DVGs or full-length genome. Consequently, DVG species with higher read numbers may encode more proteins, but without the featured fusion peptides as markers, whether there is a correlation between the abundance of DVGs identified by nanopore direct RNA sequencing and that of their encoded proteins identified by LC-MS/MS still cannot be determined. That is also the reason why we propose that, as described above, if the peptides contain discontinuous in-frame amino acid sequences derived from different portions of amino acid sequences from full-length genome-encoded proteins, or contain out-of-frame amino sequences, they are fusion peptides encoded from DVGs. Thus, at the current stage, we can only conclude that DVG can encode protein, and whether there is a correlation between the abundance of DVGs and that of their encoded proteins remains unknown. However, since the identified 6 DVGs with read number of 1 have the capability to encode proteins as determined by the current study, we can speculate that other DVG species with higher read numbers may also have the capability to encode protein although they cannot encode featured fusion peptide as markers to determine the proteins-coding capability.

It has been known that (i) coronavirus DVGs can be packaged [31], (ii) coronavirus N protein can inhibit host innate immunity [32] and (iii) innate immunity is the first line of host defense against virus infection [33]. In addition, based on the protein databases derived from the results of nanopore direct RNA sequencing in the current study, it is suggested that some DVG-encoded fusion proteins contain part or complete N protein. It is therefore speculated that one of the functions for coronavirus DVG-encoded fusion proteins is to regulate innate immunity, affecting virus replication and subsequent pathogenicity. On the other hand, coronavirus N protein has also been suggested to be important for replication and transcription (synthesis of coronavirus sgmRNAs including sgmRNA N) [34, 35]. However, N protein can only be synthesized from sgmRNA N, and consequently, the question is how coronavirus genome replicates and transcribes sgmRNAs before N protein is synthesized. As described above, because (i) coronavirus DVGs can be packaged [31], (ii) some DVGs contain partial or complete N protein ORF and (iii) DVGs can be translated as evidenced by the results of the current study, it is also argued that, after entry into the cells, the released DVGs with partial or complete N protein ORF can be immediately translated into N-containing fusion proteins, which in turn can facilitate the full-length coronavirus genome for subsequent replication and transcription before N protein is synthesized from sgmRNA N. According to the argument above, the DVG-encoded fusion proteins in coronaviruses including SARS-CoV-2 may have impact on pathogenesis through affecting innate immunity and replication. Lastly, it is also proposed that other coronavirus DVGs which encode other species of fusion proteins or out-of-frame novel proteins (when compared with the original ORFs in the full-length genome) may have different effects from those described above on pathogenesis although the functions of their encoded proteins remain to be determined. It is worth noting that, based on the previous study [26], the species and amounts of DVGs can be altered under different infection conditions such as in different infected cells and under different selection pressures. Since DVGs can encode various proteins, such alterations in the amounts and species of DVGs and thus the encoded proteins may be a way for coronavirus to respond to environmental changes, also contributing to the coronavirus pathogenesis.

The possible reasons why the featured fusion peptide was not detected in the total cell lysates by LC–MS/MS are as follows. First, because there are too many species of DVGs in cells, the amount of each DVG-encoded protein (especially the protein with the featured fusion peptide) in a fixed amount of cell lysate may not be sufficient to be detected by LC–MS/MS. Second, not every DVG-encoded protein contains the featured fusion peptides (based on the protein reference databases generated by nanopore direct RNA sequencing for BCoV), further limiting the identified number of protein species. Third, because SuperScript™ III reverse transcriptase (cat No. 18,080,044, Thermo Fisher Scientific, Waltham, USA), which is optimized to synthesize first-strand cDNA up to ~ 12 kb, was used for nanopore direct RNA sequencing, the identified coronaviral RNA species, including DVGs, may not cover all coronavirus transcripts, especially those of longer size. Thus, the protein reference databases may not contain the full information of the DVG-encoded proteins, limiting the number of protein species identified by LC–MS/MS analysis.

As shown in Figs. 1, 2 and 3, it is suggested that DVGs have the capability to encode proteins as determined by RNA–protein pull-down assay followed by LC–MS/MS. The results indicate that other DVGs may also have the capability to encode proteins. Consequently, the DVG-encoded proteins may play important roles during coronavirus infection. Thus, the current results may suggest an attractive field of study regarding the biological functions of proteins encoded by DVGs. Determining the function of DVG-encoded proteins is a priority to understand their roles in coronavirus pathogenesis. The outcomes of these studies may contribute to the development of antiviral strategies.

## Data Availability

The databases are deposited into the Open Science Framework (OSF) at https://osf.io/cm7z6/. The protein reference databases derived from nanopore direct RNA sequencing are deposited at https://osf.io/cm7z6/; file path: Data_analysis/(4) Mass_spectrometer_analysis/BCoV_cell_ORF_DVG_nanopore.xls.The databases of identified proteins by LC-MS/MS analysis are as follows. The databases for DVG-encoded proteins (total cell lysates) using protein reference databases derived from nanopore direct RNA sequencing are deposited at https://osf.io/cm7z6/; file path: Data_analysis/(4) Mass_spectrometer_analysis/BCoV-infected_HRT_total_cell_lysate/BCoV-infected_HRT_total_cell_lysate. The databases for DVG-encoded proteins (RNA-protein pull-down lysates) using protein reference databases derived from nanopore direct RNA sequencing are deposited at https://osf.io/cm7z6/; file path: Data_analysis/(4) Mass_spectrometer_analysis/BCoV-infected_HRT_with_RNA_pull-down_cell_lysate/(A) BCoV_DVG_database_result/BCoV with RPDCL by DVG. The databases for encoded proteins from human cells (RNA-protein pull-down lysates) using human protein sequences as reference databases are deposited at https://osf.io/cm7z6/; file path: Data_analysis/(4) Mass_spectrometer_analysis/BCoV-infected_HRT_with_RNA_pull-down_cell_lysate/(B) human_database_result /BCoV with RPDCL by human.

## Abbreviations

BCoV: Bovine coronavirus
CoV: Coronavirus
DVG: Defective viral genome
HRT-18: Cells, human rectal tumor-18 cells
LC-MS/MS: Liquid chromatography-tandem mass spectrometry
MOI: Multiplicity of infection
Nsp: Nonstructural protein
SARS-CoV-2: Severe acute respiratory syndrome coronavirus 2
sgmRNA: Subgenomic mRNA
UTR: Untranslated region
